# Deep Learning and Image Generator Health Tabular Data (IGHT) for Predicting Overall Survival in Patients With Colorectal Cancer: Retrospective Study

**DOI:** 10.2196/75022

**Published:** 2025-08-19

**Authors:** Seo Hyun Oh, Youngho Lee, Jeong-Heum Baek, Woongsang Sunwoo

**Affiliations:** 1 Department of IT Convergence Gachon University 1342, Seongnam-daero, Sung-nam si Republic of Korea; 2 Department of Computer Engineering Gachon University Sung-nam si Republic of Korea; 3 Division of Colon and Rectal Surgery Department of Surgery Gil Medical Center 21, Namdong-daero 774beon-gil, Incheon Republic of Korea; 4 Department of Otorhinolaryngology-Head and Neck Surgery Gachon University College of Medicine Gil Medical Center 21, Namdong-daero 774beon-gil, Incheon Republic of Korea

**Keywords:** colon, rectum, cancer, predict, prediction, predictions, predictive, model, models, health care, clinical informatics, electronic health record, EHR, South Korea, convolutional neural networks, VGG16, neural network, deep learning

## Abstract

**Background:**

Recent advances in artificial intelligence (AI) have contributed to improved predictive modeling in health care, particularly in oncology. Traditional methods often rely on structured tabular data, but these approaches can struggle to capture complex interactions among clinical variables. Image generator for health tabular data (IGHT) transform tabular electronic medical record (EMR) data into structured 2D image matrices, enabling the use of powerful computer vision–based deep learning models. This approach offers a novel baseline for survival prediction in colorectal cancer by leveraging spatial encoding of clinical features, potentially enhancing prognostic accuracy and interpretability.

**Objective:**

This study aimed to develop and evaluate a deep learning model using EMR data to predict 5-year overall survival in patients with colorectal cancer and to examine the clinical interpretability of model predictions using explainable artificial intelligence (XAI) techniques.

**Methods:**

Anonymized EMR data of 3321 patients at the Gil Medical Center were analyzed. The dataset included demographic details, tumor characteristics, laboratory values, treatment modalities, and follow-up outcomes. Clinical variables were converted into 2D image matrices using the IGHT. Patients were stratified into colon and rectal cancer groups to account for biological and prognostic differences. Three models were developed and compared: a conventional artificial neural network (ANN), a basic convolutional neural network (CNN), and a transfer learning–based Visual Geometry Group (VGG)16 model. Model performance was assessed using accuracy, sensitivity, specificity, precision, and F1-scores. To interpret model decisions, gradient-weighted class activation mapping (Grad-CAM) was applied to visualize regions of the input images that contributed most to predictions, enabling identification of key prognostic features.

**Results:**

Among the tested models, VGG16 exhibited superior predictive performance, achieving an accuracy of 78.44% for colon cancer and 74.83% for rectal cancer. It showed notably high specificity (89.55% for colon cancer and 87.9% for rectal cancer), indicating strong reliability in correctly identifying patients likely to survive beyond 5 years. Compared to ANN and CNN models, VGG16 achieved a better balance between sensitivity and specificity, demonstrating robustness in the presence of moderate class imbalance within the dataset. Grad-CAM visualization highlighted clinically relevant features (eg, age, gender, smoking history, American Society of Anesthesiologists physical status classification (ASA) grade, liver disease, pulmonary disease, and initial carcinoembryonic antigen [CEA] levels). Conversely, the CNN model yielded lower overall accuracy and low specificity, which limits its immediate applicability in clinical settings.

**Conclusions:**

The proposed IGHT-based deep learning model, particularly leveraging the VGG16 architecture, demonstrates promising accuracy and interpretability in predicting 5-year overall survival in patients with colorectal cancer. Its capability to effectively stratify patients into risk categories with balanced sensitivity and specificity underscores its potential utility as a clinical decision support system (CDSS) tool. Future studies incorporating external validation with multicenter cohorts and prospective designs are necessary to establish generalizability and clinical integration feasibility.

## Introduction

Artificial intelligence (AI) has evolved, becoming integral to various fields, including health care, bioscience, and medical diagnostics. In the medical field, AI applications range from disease detection to drug prescription optimization [1]. Health care providers use AI for patient disease prediction and data anonymization, addressing the growing health care costs associated with increasing chronic diseases and life expectancy [2]. Recent studies have increasingly applied advanced machine learning and explainable artificial intelligence (XAI) methods to improve disease survival prediction. For instance, Yang et al [3] and Yau et al [4] have demonstrated effective survival modeling using time-to-event algorithms and survival tree analyses, respectively. Huang et al [5] applied explainable machine learning approaches to uncover important risk factors across major cancers, including colorectal cancer. These studies highlight the growing trend and validate the clinical relevance of AI-driven survival prediction.

Recent advances in medical imaging technology have enabled sophisticated tumor image analysis using AI models [6]. In the field of medical image analysis, active research is being conducted using convolutional neural network (CNN) models to predict responses to treatment in patients with colorectal cancer. Yang et al [7] built a model to evaluate the risk of recurrence and metastasis by applying deep learning technology to the data of patients with benign breast cancer. Full hematoxylin and eosin (H&E)-stained images were obtained from surgical specimens of patients with breast cancer, and a CNN was applied to them. The model used in the study achieved an area under the curve (AUC) of 0.76 and showed the potential for evaluating the risk of recurrence and metastasis in patients with human epidermal growth factor receptor 2 (HER2)-positive breast cancer. Althammer et al [8] conducted a study on image analysis to predict the response to durvalumab therapy targeting the programmed cell death-1/programmed cell death ligand-1 (PD1/PD-L1) pathway in patients with non–small cell lung cancer. The results showed that the median overall survival in patients receiving durvalumab was 21 months for those positive for the CD8xPD-L1 signature and 7.8 months for those negative (*P*<.001).

In particular, the image-guided tabular data (IGTD) method proposed by Zhu et al [9] showed that arranging numerical variables into a 2D matrix format can improve model performance by enabling CNNs to capture local feature interactions. Inspired by this approach, we developed an image generator for health tabular data (IGHT) encoding method tailored to our clinical dataset, converting 25 normalized features into a 5×5 image matrix. This fixed spatial layout allows the model to process tabular information through CNN-based architectures, facilitating the use of transfer learning and potentially enhancing predictive performance. There has been growing interest in transforming structured tabular data into image representations to leverage the power of CNNs in domains traditionally dominated by machine learning. For example, Lara-Abelenda et al [10] introduced low mixed image-guided tabular data IGTD (LM-IGTD), an enhanced pipeline based on the IGTD approach. Their method applies noise-based augmentation and preserves explicit feature-to-pixel mappings, allowing for the integration of post hoc explanation techniques, such as gradient-weighted class activation mapping (Grad-CAM) and saliency maps for better interpretability. These developments reflect a broader trend toward interpretable and robust AI in clinical settings.

In this study, we built upon this direction by applying a novel tabular-to-image transformation method, IGHT, which converts structured electronic medical record (EMR) features(tabular data) into images, enabling deep CNN architectures to extract spatial patterns for survival prediction in patients with colorectal cancer.

Building upon these developments, Sharma et al [11] introduced DeepInsight, one of the pioneering frameworks that enables CNNs to process tabular data by converting them into image representations. This method uses dimensionality reduction techniques, such as t-distributed stochastic neighbor embedding (t-SNE) or principal component analysis (PCA), to spatially arrange high-dimensional features in a 2D grid, while preserving interfeature relationships. Such transformations allow CNNs to extract local and global patterns from structured data—patterns that may be overlooked by traditional machine learning approaches. This early work demonstrated the potential of tabular-to-image conversion to enhance classification performance across domains, and it has laid the foundation for subsequent advances, including this study.

AI has shown strong performance in learning from existing patient information using big data and in predicting or recommending outcomes desired by clinicians, who are key decision makers. In particular, the clinical decision support system (CDSS), an AI system that helps clinicians make decisions, has attracted considerable attention. The CDSS trains an AI model with existing medical knowledge and patient data to predict outcomes and make recommendations for clinicians based on new patient data [12]. Watson for Oncology (WFO), a representative CDSS, was developed by IBM Corp and is a system that analyzes more than 15 million pages of medical documents, 300 medical journals, and 200 guidelines [13]. The WFO was designed to recommend treatment options to oncologists and was first introduced and used in 2016 at the Gil Medical Center in Korea [14]. Although the WFO at the Gil Medical Center helped inform treatment decisions, it could not reflect regional characteristics due to differences in insurance coverage and medical costs [15].

Lee et al [16] compared the treatment recommendations generated by the WFO with the actual treatment received by 656 patients with stage 2, 3, and 4 colon cancer between 2009 and 2016 to determine the concordance rate. The results showed that the agreement rate between the WFO and the Gil Medical Center’s treatment recommendations was low, at only 48.9%. Since the WFO was trained using data from Americans patients, the prescription recommendations did not match well with those for Korean patients. In addition, it was noted that the treatment recommendations under the Korean insurance system and those under the American insurance system differed, resulting in a low concordance rate with actual treatment practices. Therefore, it is important to establish a CDSS using Korean patient data.

To build a CDSS, many studies have been conducted to enhance the performance of medical data–based AI models. Park et al [17] used an oversampling technique to address data imbalance and predicted colorectal cancer chemotherapy based on data from the Gil Medical Center in Korea using a deep learning model. Kwon et al [18] used machine learning models, such as the gradient boosted model, the distributed random forest, the generalized linear model, and the deep neural network, for a stacking ensemble. They diagnosed breast cancer using the best-performing model in the stacking ensemble. Oh et al [19] classified colorectal cancer chemotherapy regimens using machine learning models: k-nearest neighbor (kNN), support vector machine (SVM), decision tree, and light gradient boosting machine (LightGBM), and compared the results across multiple models.

Colorectal cancer is the second-most common malignancy in South Korea [20] and the second leading cause of cancer-related mortality worldwide [21]. Surgery is the primary treatment for colon and rectal cancer [22], but due to its high postsurgical mortality rate, ongoing prognosis management is essential. Therefore, research aimed at extending patient survival is being conducted from various perspectives, including the prediction of overall survival, disease-free survival, and recurrence. Overall survival and disease-free survival periods are key indicators for assessing a patient’s prognosis, and to extend life expectancy, further research is needed to evaluate prognosis based on patient-specific factors.

Studies on predicting the survival period have mainly been conducted in the clinical field using statistical techniques, such as the Kaplan-Meier method [23] and Cox’s proportional hazards model [24]. Yeom et al [25] identified prognostic factors that increase the risk of death in patients with terminal cancer and predicted the survival period according to the number of these prognostic factors. Using the Kaplan-Meier method and the log-rank test, they investigated whether there were differences in the survival period according to clinical variables. Using Cox’s proportional hazards model, they identified variables that increase the risk of death among clinical variables and used them as prognostic factors, which were then incorporated into the Weibull proportional hazards function model to predict survival periods.

**Figure 1:**
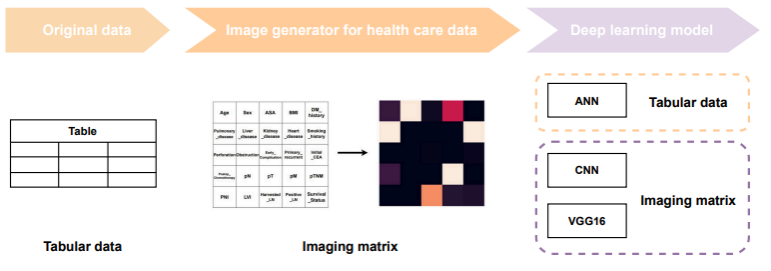
Overview pipeline of this study process. ANN: artificial neural network; ASA: American Society of Anesthesiologists physical status classification; CEA: carcinoembryonic antigen; CNN: convolutional neural network; DM: diabetes mellitus; LN: lymph nodes; LVI: lymphovascular invasion; pM: pathological distant metastasis; pN: pathological regional lymph node; PNI: perineural invasion; pT: pathological primary tumor; pTNM: pathological tumor, node, metastasis.

Figure 1 presents an overview of the pipeline of this study. In this study, we aimed to develop and evaluate deep learning–based models for predicting survival using data from patients with colorectal cancer. This study used image data by converting tabular electronic medical record (EMR) data received from the health care system into image data to enhance their utility. These deep learning models are expected to improve the performance of predicting the prognosis of patients with colorectal cancer by effectively using EMR data.

## Methods

### Dataset

In this study, EMR data were retrospectively collected from the Gil Medical Center, a tertiary referral hospital in Incheon, South Korea. A total of 3321 patients who underwent elective surgery with curative intent for colorectal cancer between 2004 and 2018 were included. The dataset was constructed through iterative chart review conducted by colorectal cancer clinicians.

Demographics are patient characteristics that can typically be known without the need for surgery. These included basic information, such as age, sex, BMI, and the patient status, as classified by the American Society of Anesthesiologists physical status classification (ASA): “DM_history” refers to a history of diabetes mellitus; “Pulmonary_disease,” a history of lung disease; “Liver_disease,” a history of liver disease; and “Kidney_disease,” a history of kidney disease.

Perioperative clinical features included information about each patient gathered before and after surgery: “Initial CEA” and “Initial Hb” represent the first blood tests performed at the time of cancer diagnosis (serum carcinoembryonic antigen [CEA] and serum hemoglobin [Hb] levels, respectively), “Transfusion_op” indicates whether a blood transfusion was performed during surgery; “Early_complication” is defined as a case of complications occurring within 30 days after surgery, and “Postop_Chemotherapy” indicates whether chemotherapy was used after surgery.

Histopathologic features can be known after a biopsy of the patient’s tumor following surgery. Here, “pTNM” is a variable that integrates the pathological tumor, node, metastasis (TNM) stage and is classified into stages 1, 2, 3, and 4. The TNM stage classification was based on the *AJCC Cancer Staging Manual, Eighth Edition*. “pT” (pathological primary tumor) includes Tis, T1, T2, T3, or T4 (T4a, T4b) as the T stage; “pN” (pathological regional lymph node) includes N0, N1 (N1a, N1b, N1c), or N2 (N2a, N2b) as the N stage; and “pM” (pathological distant metastasis) includes M1 as the M stage.

In the “Intraoperative_tumor_location” variable, colon cancer is classified into cecum, ascending colon, hepatic flexure, transverse colon, splenic flexure, descending colon, sigmoid colon, and rectosigmoid junction cancer according to the location of the primary tumor. In the case of rectal cancer, 0-5 cm of the anal verge (AV) is classified as the lower rectum, 6-10 cm of the AV is the midrectum, and 11-15 cm of the AV is the upper rectum. The final colon cancer dataset consisted of 2091 patients, and the rectal cancer dataset consisted of 1190 patients.

### Ethical Considerations

This study was reviewed and approved by the Institutional Review Board of the Gil Medical Center (GFIRB 2023-034). EMR data from the Gil Medical Center were used. Overall, data of 3321 patients were retrospectively collected through an iterative chart review conducted by colorectal cancer specialists. Due to its retrospective nature, the study was exempt from requiring informed consent from the participants.

### Data Preprocessing

The variables were selected in consultation with a clinician. To select and use clinical variables to enhance the explanatory power of the results, pretreatment was performed after discussion with the clinician. Patient exclusion, variable categorization, missing value removal, and variable selection were performed in that order. All continuous variables were normalized to a (0,1) scale using min-max normalization to standardize the color intensity across features. Categorical variables were first one-hot-encoded and then similarly mapped to the image matrix, ensuring consistent scaling across data types during the image generation process. Images were constructed by arranging features sequentially based on the column order in the original dataset. No domain-driven grouping or clustering of related variables was applied to preserve reproducibility and avoid introducing subjective bias. Although the spatial arrangement of semantically related features may potentially affect CNN performance, this aspect was not explored in the study and is left for future investigation.

Prior to variable selection, patient cases excluded from the analysis were removed. In the case of “Kidney_disease,” missing values were deleted. Patients who underwent surgery for recurrent colorectal cancer were also excluded.

After excluding patients, variable categorization was performed. Age was categorized by the number 65, which is a standard used to divide age. The BMI was categorized as 18 or less and 18 or more and as 25 or less and 25 or more. In addition, “DM_history,” “Pulmonary_disease,” “Liver_disease,” and “Heart_disease,” which are variables corresponding to the patient’s medical history, were categorized by the presence or absence of a medical history.

Among the perioperative clinical features, “Initial_CEA,” which indicates the first CEA level after diagnosis, was categorized according to 5 criteria, and 166 missing values were replaced with the average value. Missing values were removed for “Early_Complication,” which indicates complications within 30 days after surgery. In particular, “Overall_Survival” was calculated from the date of surgery until the date of death for uncensored cases or until the date of last follow-up for censored cases. In addition, patients were categorized based on 5 years (60 months), which is the criterion for cure.

Among histopathologic features, missing values were removed and categorized in “Havested_LN,” which represents the number of lymph nodes removed from the patient during surgery. If more than 12 pieces were removed, the operation was considered successful, and if less than 12 pieces were removed, the operation was classified as insufficient. Missing values were also removed from “Positive_LN,” which indicates the number of metastatic lymph nodes that could be recognized during surgery. The final selected variables are listed in Table S1 in Multimedia Appendix 1.

### Image Generation Health Care Tabular Data Method

**Figure 2:**
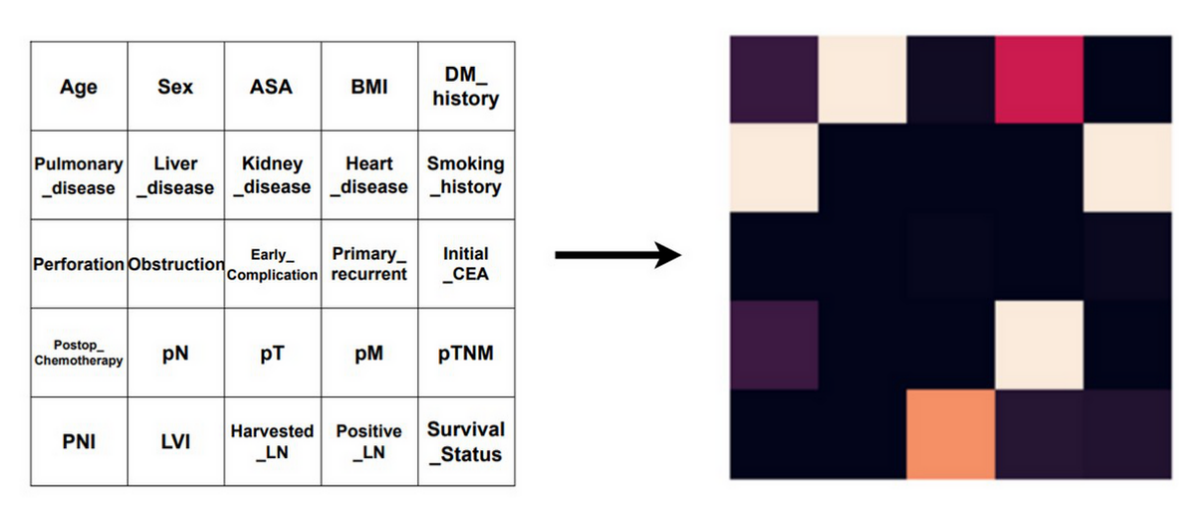
Method of generating IGHT. ASA: American Society of Anesthesiologists physical status classification; CEA: carcinoembryonic antigen; DM: diabetes mellitus; IGHT: image generator health care tabular data; LN: lymph nodes; LVI: lymphovascular invasion; pM: pathological distant metastasis; pN: pathological regional lymph node; PNI: perineural invasion; pT: pathological primary tumor; pTNM: pathological tumor, node, metastasis.

In this study, we aimed to convert the data from structured data into 2D unstructured image data to use as input to the deep learning model. The variables of the tabular data were visualized in matrix form and developed according to the data. Each variable in Table S1 in Multimedia Appendix 1 was mapped to one column of the heat map and implemented in the form of an image. Figure 2 shows an example of an imaging matrix. The image is in the form of N×N, where N is 5, and the number of variables used for image conversion is 25.

In this study, we selected 25 clinically relevant variables and reshaped them into a 5×5 matrix to generate 2D images for input into the CNN models. Variables were categorized into three clinically meaningful groups: demographic, perioperative, and histopathologic features. These variables were mapped from left to right and top to bottom in the 5×5 matrix, following the order of these categories. This spatial configuration was carefully designed to enable the deep learning model to effectively capture interactions among clinically related variables. By organizing the input into a square-shaped matrix, the convolutional filters in the CNN architecture could exploit spatial proximities from the input layer, thereby enhancing the model’s ability to learn joint patterns and improving both performance and interpretability.

We also considered scenarios where the number of input features may not perfectly form a square matrix. In such cases, possible strategies include (1) zero-padding to fill the remaining space without introducing clinical meaning and (2) modifying the CNN input layer to accept nonsquare (rectangular) input dimensions. Although these were not necessary in this study, they remain relevant for future extensions of this framework.

### Deep Learning Model Prediction

**Figure 3:**
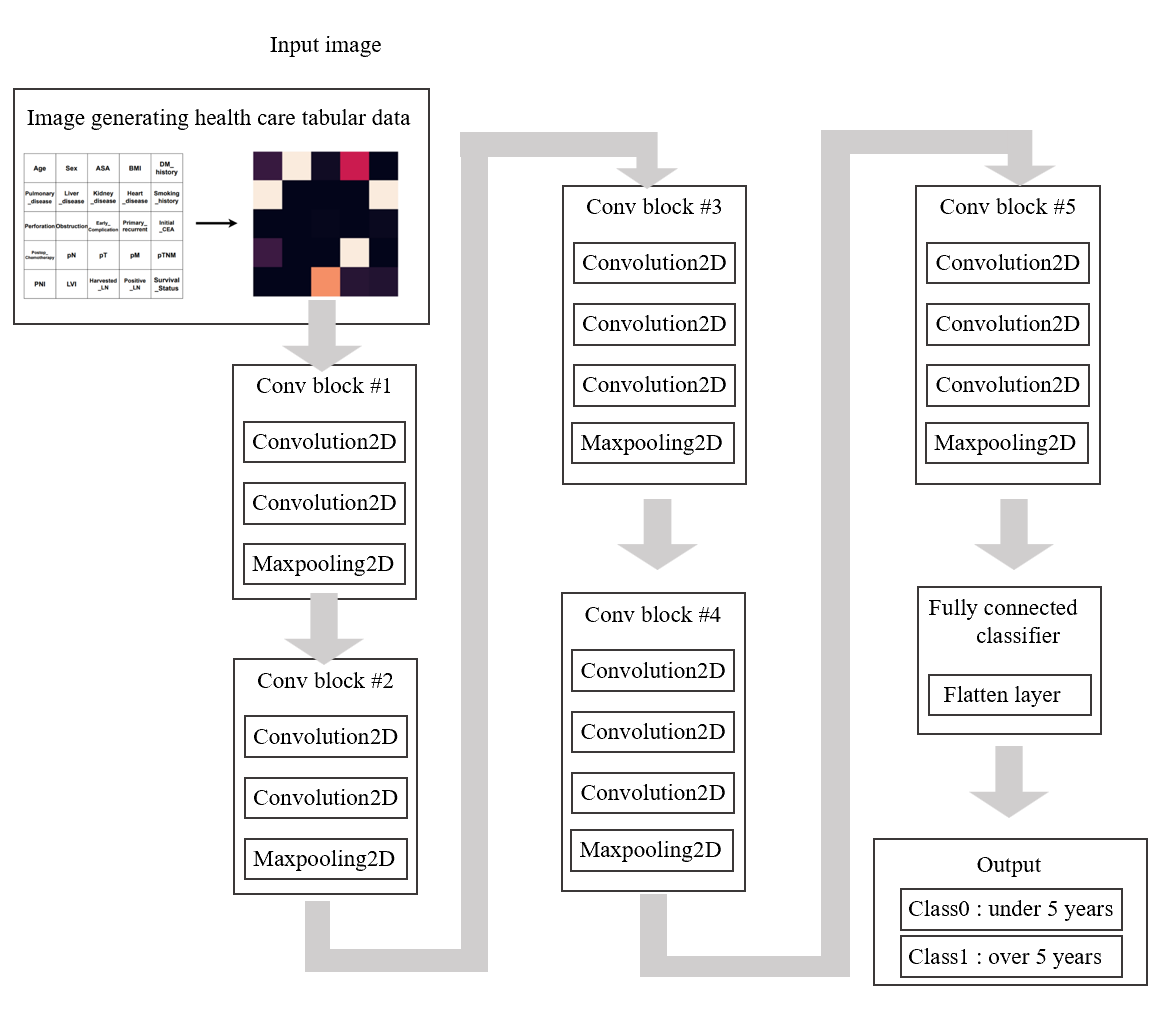
Pipeline of VGG16 using the IGHT technique. ASA: American Society of Anesthesiologists physical status classification; CEA: carcinoembryonic antigen; DM: diabetes mellitus; IGHT: image generator for health tabular data; LN: lymph nodes; LVI: lymphovascular invasion; pM: pathological distant metastasis; pN: pathological regional lymph node; PNI: perineural invasion; pT: pathological primary tumor; pTNM: pathological tumor, node, metastasis; VGG: Visual Geometry Group.

Figure 3 shows a pictorial representation of the VGG16 model, proposed by the Visual Geometry Group (VGG), used in this study. The weights of VGG16 learned using a large dataset called ImageNet were imported and used to classify the imaging matrix. All modeling and analysis were conducted using Google Colaboratory with the following software environment: Python 3.7.13, TensorFlow 2.8.0, Keras 2.8.0, pandas 1.3.5, scikit-learn 1.0.2, NumPy 1.21.5, matplotlib 3.4.3, and seaborn 0.11.2.

For model interpretation, Grad-CAM was implemented using custom code adapted from tf-keras-vis and compatible TensorFlow visualization utilities. We divided the dataset of 3321 patients with colorectal cancer into two cohorts—colon cancer (n=2089, 62.9%) and rectal cancer (n=1232, 37.1%)—and trained separate models for each group. For both cohorts, the data were randomly split into training and testing sets in a 7:3 ratio.

ANN and CNN models were trained using default hyperparameters. For the VGG16-based model, we adopted a transfer learning approach: the convolutional base of VGG16 pretrained on ImageNet was used (include_top=False), and all layers were set to be trainable. A flattened output was passed through a dense layer with 256 units (rectified linear unit [ReLU] activation), followed by a dropout layer (rate=0.25) and a sigmoid output layer for binary classification. The optimizer used was Adam with a learning rate of 0.01, and the loss function was binary cross-entropy. We also tested the Stochastic Gradient Descent (SGD) optimizer during model development, but it yielded inferior performance compared to Adam. Key hyperparameters, such as optimizer type, learning rate, and dropout rate, were selected based on empirical validation performance, rather than being used as default values.

Model performance was evaluated using the testing set, and the metrics reported included accuracy, sensitivity, and specificity.

#### ANN Model

After converting the tabular data into an imaging matrix, an ANN was constructed to compare and verify the AI model results. Instead of using the data converted to the imaging matrix (tabular data), the original dataset was used. Using the original dataset, an ANN was used to predict the survival period of patients with colorectal cancer and validate the results. Table S2 in Multimedia Appendix 1 lists the parameters of the baseline model in detail.

#### CNN Model

In this study, after transformation of data into an imaging matrix, a CNN model was applied. The model was constructed to classify the data by dividing the survival period of patients with colorectal cancer by 5 years. The classification performance of the patient survival period was confirmed using the CNN model, which showed good performance in image classification. Table S3 in Multimedia Appendix 1 lists the parameters of the CNN model.

#### VGG16 Model

**Figure 4:**
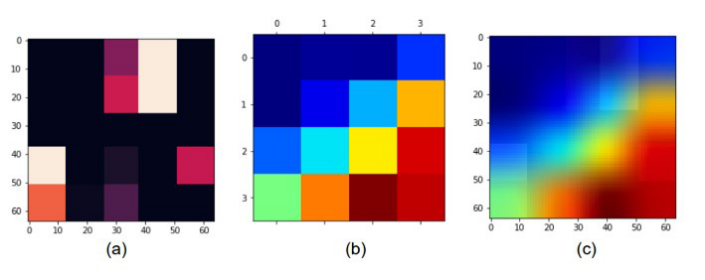
Example of Grad-CAM results: (a) original data from the image generator, (b) heat map produced by Grad-CAM, and (c) visualized image generated by Grad-CAM. Grad-CAM: gradient-weighted class activation mapping.

In this study, transfer learning based on the VGG16 model created by Simonyan and Ziserman in 2014 was performed. The VGG16 model is a structure developed from the existing CNN structure, and because it is convenient to apply, studies using VGG16 are actively being conducted to improve classification performance. Table S4 in Multimedia Appendix 1 shows the structure of VGG16 used in this study. Figure 4 shows a pictorial representation of the transfer learning method used in this study. In this study, the weights of VGG16 learned using a large dataset called ImageNet were imported and used to classify the imaging matrix.

### Gradient-Weighted Class Activation Mapping

To demonstrate the models’ potential as a CDSS, we used Grad-CAM [26] to visualize important features for individual patient predictions. VGG16 is a CNN-based model that cannot explain the results of a model [27]. Therefore, Grad-CAM was used to increase the explanatory potential of the model results. Grad-CAM was used to generate heat maps that highlighted variables with notable influence on the models’ prediction of the survival period of patients with colorectal cancer. The heat maps generated by Grad-CAM are highlighted in red and are a method of displaying the variable area that has a large influence on the prediction. Grad-CAM extracts features using weights extracted from the last convolutional layer of the models used for prediction [28].

An example of converting Grad-CAM is shown in Figure 4. In the weight of the models trained using patient data (Figure 4a), the influence of the variable that affected the prediction result was converted into a heat map of the form in Figure 4b. Next, an image was created in a form that could intuitively determine the influence, as shown in Figure 4c, overlaid on Figure 4a.

### Evaluation Metrics

The predictive performance of the models was evaluated using three key metrics: accuracy, sensitivity, and specificity.

Accuracy measures the overall proportion of correctly classified instances. Sensitivity (also known as recall) quantifies the proportion of true-positive cases correctly identified by the model, while specificity measures the proportion of true negatives correctly identified.

These metrics were calculated on the test datasets to assess the generalization performance of the models.

## Results

### Imaging Matrix Transformation

**Figure 5:**
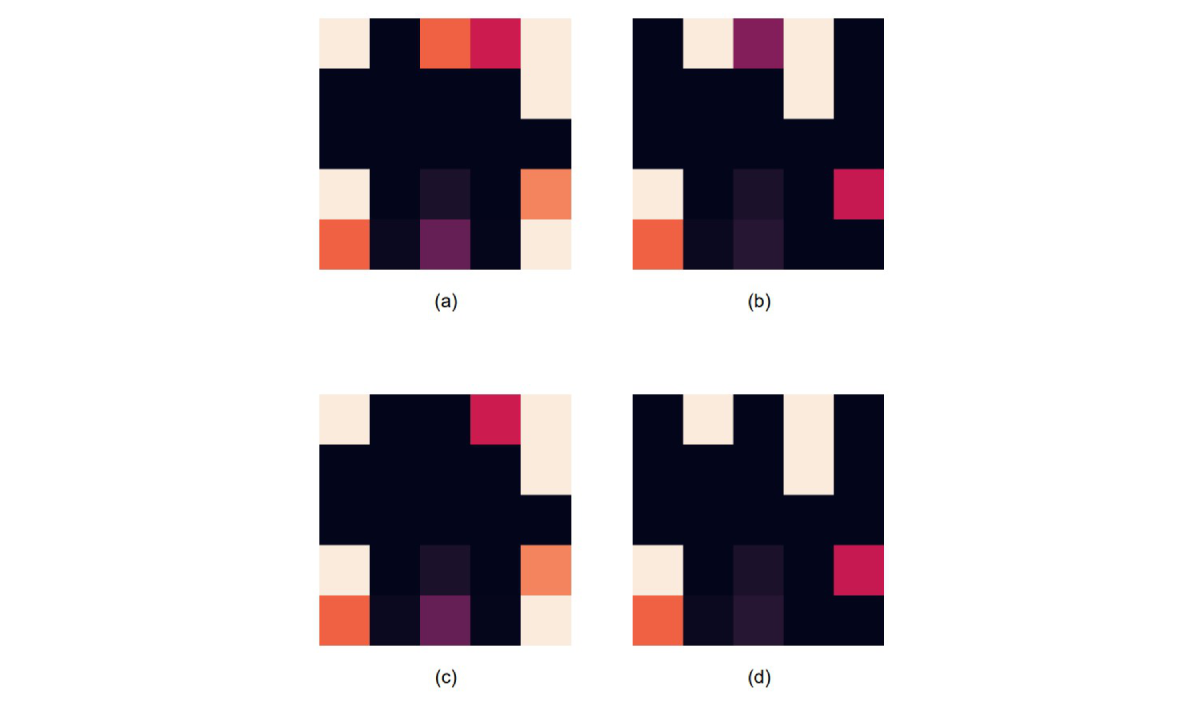
Example of an imaging matrix: (a) class 0 image in colon cancer, (b) class 1 image in colon cancer, (c) class 0 image in rectal cancer, and (d) class 1 image in rectal cancer.

The transformation of tabular data into 5×5 imaging matrices using 25 variables from patients with colorectal cancer demonstrated distinctive visual patterns. The heat map representation, where values approaching 1 appear white and those approaching 0 appear black, provided an intuitive visualization of patient data. For survival period prediction, we established a binary classification. Figure 5 shows an example of the imaging matrix created. Class 0 represented patients with a survival period of less than 5 years, while class 1 represented patients with a survival period of more than 5 years.

### Comprehensive Model Performance Evaluation

We conducted an extensive comparative analysis across three distinct deep learning architectures, each revealing unique strengths in colorectal cancer survival prediction.

As shown in Tables 1 and 2, the accuracy and specificity of VGG16 were the best among the three models. However, the sensitivity of VGG16 was the lowest among the three models, which means that the model mistakenly thought that the actual survival period of a patient was less than 5 years but that the patient had a good prognosis. Compared to VGG16, the ANN and CNN showed higher sensitivities (84%-99%) in identifying patients with poor prognoses well. However, in terms of specificity, VGG16 showed better performance in correctly predicting patients with a 5-year survival period (89.55% for colon cancer and 87.9% for rectal cancer).

**Table 1:** Overall survival prediction in patients with colon and rectal cancer using ANN^a^, CNN^b^, and VGG16^c^ models.

Model	Accuracy (%)	Sensitivity (%)	Specificity (%)	Precision (%)	*F*_1_-score (%)
ANN	71.47	84.00	54.23	71.59	76.86
CNN	61.90	99.40	4.90	61.58	76.17
VGG16	78.23	40.79	89.55	80.55	54.33

^a^ANN: artificial neural network.

^b^CNN: convolutional neural network.

^c^VGG: Visual Geometry Group.

**Table 2:** Overall survival prediction in patients with rectal cancer using ANNa, CNNb, and VGG16c models.

Model	Accuracy (%)	Sensitivity (%)	Specificity (%)	Precision (%)	*F*_1_-score (%)
ANN	74.78	87.46	55.26	74.14	80.18
CNN	61.80	97.71	4.93	61.35	75.81
VGG16	76.54	35.56	87.90	78.68	49.02

^a^ANN: artificial neural network.

^b^CNN: convolutional neural network.

^c^VGG: Visual Geometry Group.

### Gradient-Weighted Class Activation Mapping

Grad-CAM analysis was used to enhance the interpretability of the VGG16 model predictions. This technique generated heat maps highlighting variables that appeared most influential in the three models’ survival period predictions.

#### Analysis of Patients With Colon Cancer

**Figure 6:**
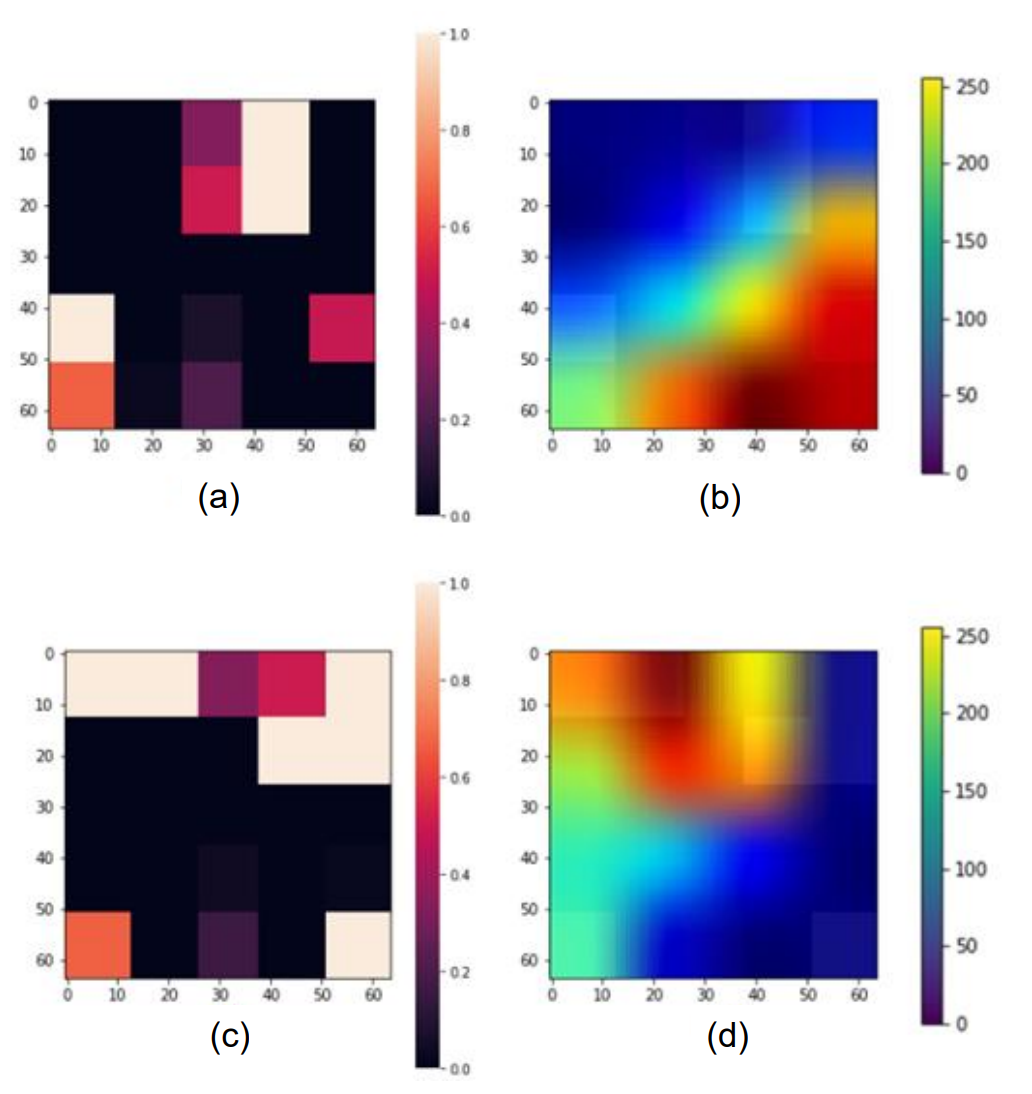
Visualized results generated by Grad-CAM in patients with colon cancer: (a, c) original data from the image generator and (b, d) visualized image generated by Grad-CAM. Grad-CAM: gradient-weighted class activation mapping.

Figure 6 presents examples of heat map conversion results and Grad-CAM application results for patients with colon cancer. The patient has a survival period of more than 5 years and is a successful case of deep learning model prediction. Looking at Figure 6a, the patient was in an early pTNM stage. Considering this, it can be confirmed that the factor that played a major role in VGG16 predicting the survival of this patient was the pTNM stage. The results of Grad-CAM for patients without liver disease and a moderate ASA grade had an impact on predicting that patients would have longer survival times (Figure 6d).

#### Analysis of Patients With Rectal Cancer

**Figure 7:**
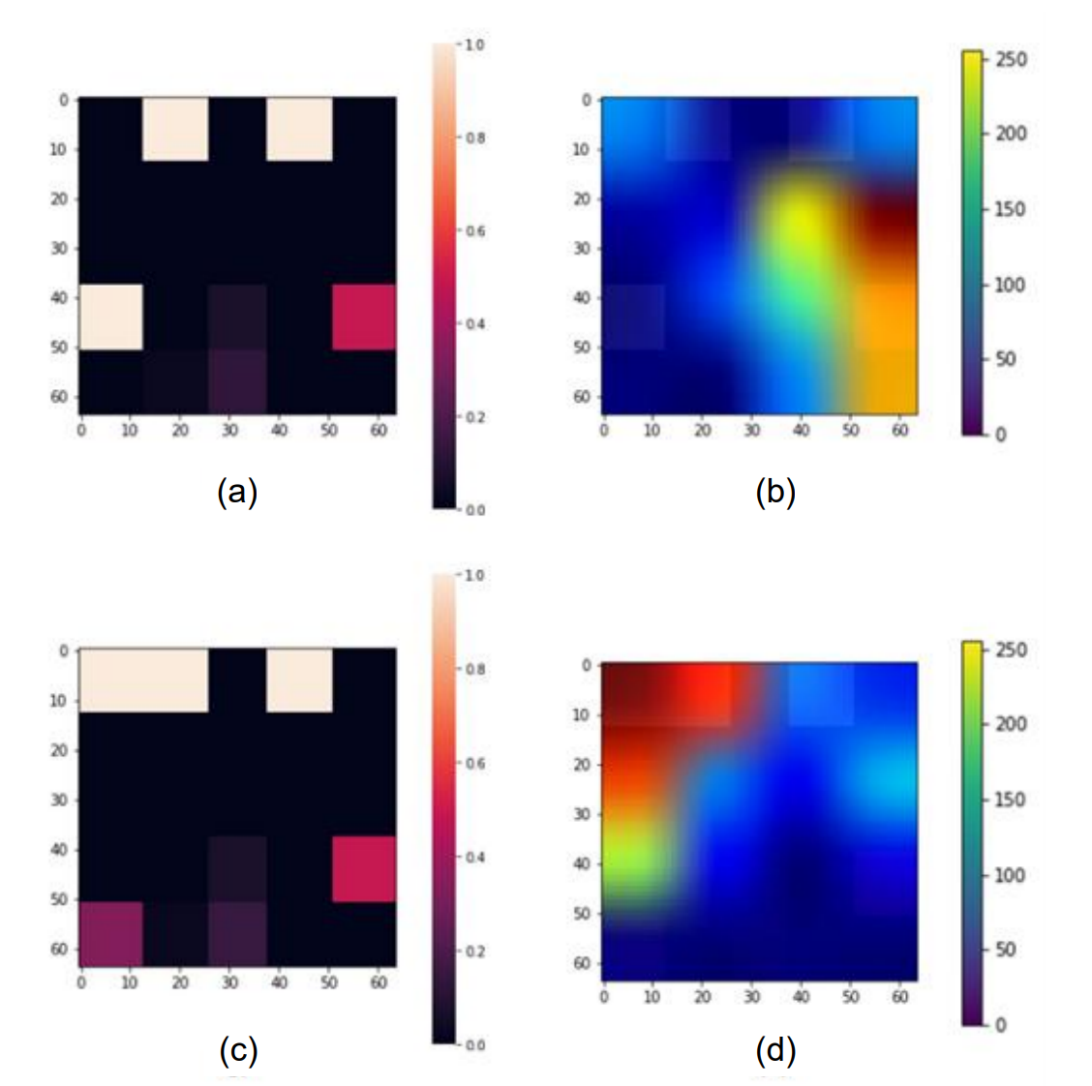
Visualized results generated by Grad-CAM in patients with rectal cancer: (a, c) original data from the image generator and (b, d) visualized image generated by Grad-CAM. Grad-CAM: gradient-weighted class activation mapping.

Figure 7 presents examples of heat map conversion results and Grad-CAM application results for patients with rectal cancer. Figures 7a and 7c are the learned images, and Figures 7b and 7d show the results of Grad-CAM as a heat map. As shown in the imaging matrix in Figure 7b, the smoking history, initial CEA level, and pTNM stage were indicated as variables that had a major influence on the prediction of the model: there was no smoking history, the initial CEA level was moderate, and the TNM stage was early. As the patient’s survival period was well predicted to be more than 5 years, it was found that the Grad-CAM analysis results were consistent with the results of existing clinical studies. As shown in Figure 7d, age, sex, the ASA grade, and pulmonary disease affected the survival time prediction results. For a patient with a high age, a low ASA grade, and no pulmonary disease, with a well-predicted survival of more than 5 years, because the ASA grade was low, the patient’s physical condition was good, and there was no pulmonary disease, the survival period seemed to be predicted for a long time. As described earlier, Grad-CAM can be used to determine which variables have the most influence on the prediction of survival time for individual patients and their risk factors.

## Discussion

### Principal Findings

In this study, we developed a model to predict the survival period of colorectal cancer using EMR data and investigated which variables contributed to the prediction. In particular, we improved the performance of the model through an innovative approach to convert tabular medical data into image data. The results of the study showed that the VGG16 model achieves the best performance, which suggests a new methodology for developing a CDSS for patients with colorectal cancer in clinical settings.

Deep learning models performed important clinical predictions on whether the survival period of patients with colorectal cancer who underwent surgery is more than 5 years. If the survival period of a patient with cancer is more than 5 years, it indicates that the patient’s prognosis is good and the risk of cancer recurrence is low, which can provide clinically helpful information. Furthermore, it shows that doctors can use this as a reference to understand the patient’s condition and support them in making better decisions.

In this study, the prediction accuracy of VGG16 was 75%, and that of the CNN was 61%, showing that the VGG16 model, which used more weights through transfer learning, performs better. The reason for the large performance difference between the CNN and VGG16, about 14%, is probably because the amount of data used in this study was less than that used in other deep learning models. However, VGG16 is a transfer learning model trained using a large ImageNET-based dataset and provides the weights obtained as a result. In this study, we used the weights of VGG16 for learning, and we were able to see that the prediction performance was improved by overcoming the limitations of quantitative data volume.

Given the class imbalance in our dataset and the differing behavior observed across models, a clear understanding of the trade-off between sensitivity and specificity is essential for clinical application. Although oversampling techniques, such as the synthetic minority oversampling technique (SMOTE), were initially considered to mitigate the imbalance, we ultimately decided not to apply them after consultation with clinical experts. It was determined that artificially augmenting the minority classes could compromise the clinical validity and real-world representativeness of colorectal cancer data. Therefore, we maintained the original class distribution and focused on evaluating model performance through sensitivity and specificity, which are more aligned with clinical priorities. To further enhance model interpretability and support clinical decision-making, we applied Grad-CAM to visualize the features contributing to model predictions.

Our best-performing model, VGG16, exhibited high specificity (88%-90%) but relatively low sensitivity (35%-40%). This indicates that when the model predicts early mortality (nonsurvival within 5 years), it is highly reliable, minimizing false positives in identifying high-risk patients. Such high specificity is valuable in clinical settings where unnecessary aggressive follow-up could impose physical, psychological, and economic burdens. Considering the class distribution (approximately 6:4 for positive to negative outcomes, where “positive” refers to patients surviving for more than 5 years), precision and *F*_1_-scores provide further insight into model behavior. VGG16, which showed the highest specificity and precision, tended to make more conservative predictions regarding long-term survival, resulting in fewer false positives. From a CDSS perspective, its relatively high *F*_1_-score and sensitivity suggest potential usefulness in identifying patients unlikely to survive beyond 5 years. Although the ANN and CNN also demonstrated comparable sensitivity, VGG16 maintained this without compromising specificity, indicating a more balanced performance that may help reduce false alarms in clinical practice.

The VGG16 model outperformed the ANN model by achieving an accuracy improvement of 6.76% (78.23% vs 71.47%) in colon cancer survival prediction and 1.76% (76.54% vs 74.78%) in rectal cancer survival prediction. Additionally, VGG16 demonstrated markedly higher specificity compared to the ANN (89.55% vs 54.23% for colon cancer and 87.9% vs 55.26% for rectal cancer), indicating improved ability to correctly identify patients with better survival outcomes.

In contrast, the CNN model showed substantially lower accuracy (61.9% for colon cancer and 61.8% for rectal cancer) and poor specificity (<5%), suggesting limited clinical utility in its current form. These findings highlight that although basic CNN architectures may underperform, deeper networks, such as VGG16, can capture complex patterns to improve prediction reliability.

However, the lower sensitivity implies that some patients who do die early are not identified by the model (false negatives), potentially missing individuals who could benefit from intensified monitoring or interventions. This limitation is crucial to acknowledge, as underdetection may reduce the model’s effectiveness in guiding proactive clinical decision-making.

Conversely, the CNN model showed the opposite pattern, with high sensitivity but low specificity, which would result in many false alarms and potentially excessive interventions.

Therefore, the choice of model and classification threshold must be carefully tailored to the clinical context and intended use case. For example, a model prioritizing sensitivity may be preferred in screening scenarios to ensure at-risk patients are not missed, whereas one prioritizing specificity may be favored where reducing false positives is paramount.

Unlike the black-box model, the demand for a white-box model that provides a reason for the result is increasing [29]. A white-box model explains the results of an AI model and has recently been attracting attention under the name of XAI [30].

Although our model achieved an accuracy of 75%-78% in predicting 5‑year survival, the existing literature indicates that this level of performance is clinically meaningful. Kos et al [31] used various machine learning models (eg, decision tree, stacking ensemble, and SVM) to predict survival rates from 1 to 10 years using large-scale data of patients with colorectal cancer in Australia. In this study, the 5-year survival prediction models showed an AUC of approximately 0.86-0.89 and an accuracy of over 70%, and the performance by cancer stage also showed an excellent predictive power of over 70%. Compared to the prediction accuracy of this study (78.4% for colon cancer and 74.8% for rectal cancer), both studies suggest that machine learning–based clinical data use is effective for survival prediction, and this study is particularly different in that it converted EMR data into images and applied them to a CNN-based model. Similarly, Gao et al [32] compared nine ML models against TNM staging alone (AUC=0.784) and found that most models only modestly outperform staging, often with overlapping CIs.

These findings suggest that our image‑based deep learning model operates within a comparable performance band, while adding the benefit of interpretable Grad‑CAM visualization. Furthermore, staging‑only approaches often fall short when applied to patients with stage II-III cancer, whose outcomes are less deterministic. Our model showed improved discrimination in this subgroup, indicating its potential to complement staging heuristics in borderline clinical cases.

Finally, the alignment between Grad‑CAM highlighting (eg, pTNM stage, smoking history, age) and established prognostic factors enhances the model’s trustworthiness and suitability for integration into explainable CDSSs. To help clinical decision-making, it is better for deep learning models to also provide reasons for their decisions. In this study, Grad-CAM was used to provide XAI in deep learning models. Grad-CAM can answer the reasons for individual patient outcomes, and the survival prediction model shows the possibility of providing tailored medicine as part of a CDSS.

### Clinical Implications

Figures 6 and 7 show the results of analyzing the results for some patients with colorectal cancer using Grad-CAM. Among the patients with colon and rectal cancer, we randomly selected 2 patients and examined the heatmap from the Grad-CAM analysis results. As a result, the pTNM stage was found to be the variable that had the greatest influence on the model prediction in both cancer types. However, additional variables that affected each patient were different: age, gender, smoking history, ASA grade, liver disease, pulmonary disease, and initial CEA levels. The results of Grad-CAM analysis for patients in Figure 5d,e show that the pTNM stage has an influence on the prediction, which is consistent with existing medical knowledge [33]. Figure 6 shows that smoking history has a major influence on the prediction by the model. In existing clinical studies, smoking history has been identified as a relevant factor affecting patient survival [34]. Since various factors must be considered in order to determine the prognosis of patients with colon cancer, if we conduct an analysis that can identify the influential variables at once, such as Grad-CAM, we will be able to provide better clinical services.

### Limitations and Future Work

When training a deep learning model using a small amount of data, overfitting may occur. If overfitting occurs, predictions may not work well with data other than trained data. Therefore, if we can collect more multicenter studies or data in future research, we will further increase the reliability of the study by conducting an analysis by stage.

To leverage the spatial learning capabilities of CNNs, we transformed 25 clinical variables into a 5×5 image for each patient. Each feature was min-max-normalized and assigned to a fixed position in the 5×5 matrix, ensuring consistent spatial encoding across all samples. Although clinical tabular data do not possess an inherent spatial structure, prior studies have shown that imposing a structured layout allows CNNs to effectively capture local feature interactions and complex nonlinear relationships that may be overlooked by traditional models. This image-based representation also enables the application of transfer learning using pretrained CNN architectures.

Although the variable layout was arbitrarily fixed in this study, future work may explore data-driven arrangements based on feature correlation or clinical relevance to further enhance the model’s representational capacity.

The proposed model, although demonstrating strong predictive performance, must address several practical considerations to be implemented as a CDSS. Integration into existing EMR systems would require standardized data pipelines, interoperability, and real-time processing capabilities. Additionally, interpretability remains essential for clinical adoption. In this study, we used Grad-CAM to identify features contributing most to each prediction, providing visual explanations that could be embedded into future CDSS interfaces.

From a clinical perspective, prediction models for survival outcomes in colorectal cancer can serve as valuable tools, particularly in the postoperative setting. Previous studies have demonstrated the utility of machine learning for guiding surveillance and adjuvant therapy decisions [35,36]. Our deep learning–based model offers automated and objective risk stratification using routinely collected clinical data, supporting multidisciplinary teams in identifying high-risk patients. Within clinical workflows, the system could deliver actionable alerts—such as notifications flagging patients with poor predicted survival—to prompt timely follow-up or intervention. These alerts, coupled with interpretable Grad-CAM visualizations, can enhance clinical reasoning, build trust, and facilitate shared decision-making by clearly communicating risk to both clinicians and patients.

Although this study adopted a binary classification approach (predicting 5-year survival), we acknowledge that this represents a simplification of the underlying clinical reality. In oncology, the timing of events such as recurrence or mortality is often just as important as whether the events occur. Therefore, future work will explore modeling survival outcomes in a time-to-event framework using deep learning–based survival models. Methods such as DeepSurv, a Cox proportional hazards–based neural network, have shown promise in capturing complex nonlinear relationships, while preserving the structure of survival data [37]. These approaches could yield more clinically informative predictions, especially in patient-level prognostication and individualized treatment planning.

In addition, future enhancements could include the use of more advanced convolutional architectures, such as ResNet or EfficientNet, as well as ensemble learning strategies. These techniques may help improve both performance and generalizability, especially in heterogeneous clinical datasets.

The dataset used in this study was built through the EMR system of a single institution. For multi-institutional research, if a standardized medical information system for each type of medical institution in Korea is established and advanced, it will be possible to produce generalized prediction results using more patient data. Furthermore, if data construction standards for each institution are established, it is believed that the establishment of a data mart will help Korea’s data ecosystem and contribute to the generalization of research results.

The dataset used in this study was limited as it was collected from a single institution. Therefore, the characteristics of the patient group in this study may not reflect the clinical features of patients in other medical institutions. In future research, we will present a generalized model that reflects the clinical features of patients in a multicenter study.

### Conclusion

XAI research that enhances the interpretability of deep learning model results is actively progressing. In this study, we used the IGHT technique to convert structured data into images, which allowed us to capture relationships between variables effectively. Using this approach, we developed models to predict overall survival, a key indicator for determining the prognosis of patients with colorectal cancer after surgery.

An ANN model, a CNN model, and transfer learning with a pretrained VGG16 model were used to evaluate predictive performance. Our results suggest that image-based input leads to improved prediction compared to traditional tabular data analysis. Additionally, CNN-based models provide opportunities for enhanced interpretability through techniques such as Grad-CAM.

## Appendix

Multimedia Appendix 1 Feature overview of the dataset, parameters of the artificial neural network (ANN) model, parameters of the convolutional neural network (CNN) model, and parameters of the Visual Geometry Group (VGG)16 model.

## Abbreviations

AI: artificial intelligence
ANN: artificial neural network
ASA: American Society of Anesthesiologists physical status classification
AUC: area under the curve
AV: anal verge
CDSS: clinical decision support system
CEA: carcinoembryonic antigen
CNN: convolutional neural network
DM: diabetes mellitus
EMR: electronic medical record
Grad-CAM: gradient-weighted class activation mapping
IGHT: image generator health care tabular data
IGTD: image-guided tabular data
pM: pathological distant metastasis
pN: pathological regional lymph node
pT: pathological primary tumor
pTNM: pathological tumor, node, metastasis
PNI: perineural invasion
LN: lymph nodes
LVI: lymphovascular invasion
SVM: support vector machine
TNM: tumor, node, metastasis
VGG: Visual Geometry Group
WFO: Watson for Oncology
XAI: explainable artificial intelligence
