# Supplementary material for: Deep Learning and Image Generator Health Tabular Data (IGHT) for Predicting Overall Survival in Patients With Colorectal Cancer: Retrospective Study
Source: JMIR Med Inform. 2025 Aug 19;13:e75022. doi: 10.2196/75022 (PMC12405791; doi:10.2196/75022)
Supplement: Multimedia Appendix 1 [file medinform_v13i1e75022_app1.docx]

**Appendix 1**

Deep Learning and IGHT (Image Generater Health Tabular data) for Predicting Colorectal Cancer Patients Overall Survival

**Table of Contents Page (s)**

**Supplementary Table S1.** Feature overview of dataset 2

**Supplementary Table S3.** Parameters of ANN model 3

**Supplementary Table S3.** Parameters of CNN model 4

**Supplementary Table S4.** Parameters of VGG16 model 5

**Supplementary table S1. Feature overview of dataset**

| **Category** | **Features** |
| --- | --- |
| Demographic features | Age, Sex, BMI, ASA, DM_history, Pulmonary_disease, Liver_disease, Heart_disease, Kidney_disease,  Smoking_history |
| Perioperative clinical  features | Initial_CEA, Early_Complication, Primary_recurrent, Postop_Chemotherapy, Survival_status, Overall_Survival |
| Histopathologic features | LVI, PNI, pT, pN, pM, pTNM, Perforation, Obstruction, Harvested_LN, Positive_LN |

**Supplementary table S2. Parameters of ANN model**

| **Layer** | **Output Shape** | **Param #** |
| --- | --- | --- |
| Dense | (None, 10) | 260 |
| Dense | (None, 14) | 154 |
| Dense | (None, 1) | 15 |

**Supplementary table S3. Parameters of CNN model**

| **Layer** | **Output Shape** | **Param #** |
| --- | --- | --- |
| Convolution 2D | (None, 62, 62, 16) | 448 |
| Max pooling 2D | (None, 31, 31, 16) | 0 |
| Convolution 2D | (None, 29, 29, 32) | 4640 |
| Max pooling 2D | (None, 14, 14, 32) | 0 |
| Convolution 2D | (None, 12, 12, 32) | 9248 |
| Max pooling 2D | (None, 6, 6, 32) | 0 |
| Flatten | (None, 1152) | 0 |
| Dense | (None, 512) | 590336 |
| Dense | (None, 1) | 513 |

**Supplementary table S4. Parameters of VGG16 model**

| **Layer** | **Output Shape** | **Param #** |
| --- | --- | --- |
| Input | (None, 64, 64, 3) | 0 |
| Convolution 2D | (None, 62, 62, 64) | 1792 |
| Convolution 2D | (None, 62, 62, 64) | 36928 |
| Max pooling 2D | (None, 32, 32, 64) | 0 |
| Convolution 2D | (None, 32, 32, 128) | 73856 |
| Convolution 2D | (None, 32, 32, 128) | 147584 |
| Max pooling 2D | (None, 16, 16, 128) | 0 |
| Convolution 2D | (None, 16, 16, 256) | 295168 |
| Convolution 2D | (None, 16, 16, 256) | 590080 |
| Convolution 2D | (None, 16, 16, 256) | 590080 |
| Max pooling 2D | (None, 8, 8, 256) | 0 |
| Convolution 2D | (None, 8, 8, 512) | 1180160 |
| Convolution 2D | (None, 8, 8, 512) | 2359858 |
| Convolution 2D | (None, 8, 8, 512) | 2359858 |
| Max pooling 2D | (None, 14, 14, 32) | 0 |
| Convolution 2D | (None, 4, 4, 512) | 2359858 |
| Convolution 2D | (None, 4, 4, 512) | 2359858 |
| Convolution 2D | (None, 4, 4, 512) | 2359858 |
| Max pooling 2D | (None, 2, 2, 512) | 0 |
| Flatten | (None, 2046) | 0 |
